# Supplementary material for: Novel Driver Strength Index highlights important cancer genes in TCGA PanCanAtlas patients
Source: PeerJ. 2022 Aug 11;10:e13860. doi: 10.7717/peerj.13860 (PMC9375969; doi:10.7717/peerj.13860)
Supplement: Supplemental Information 2 [file peerj-10-13860-s002.zip › COHORTS/genes plots/detailed/2021_8_16_14_9_distribution_events_detailed_females_43.pdf]

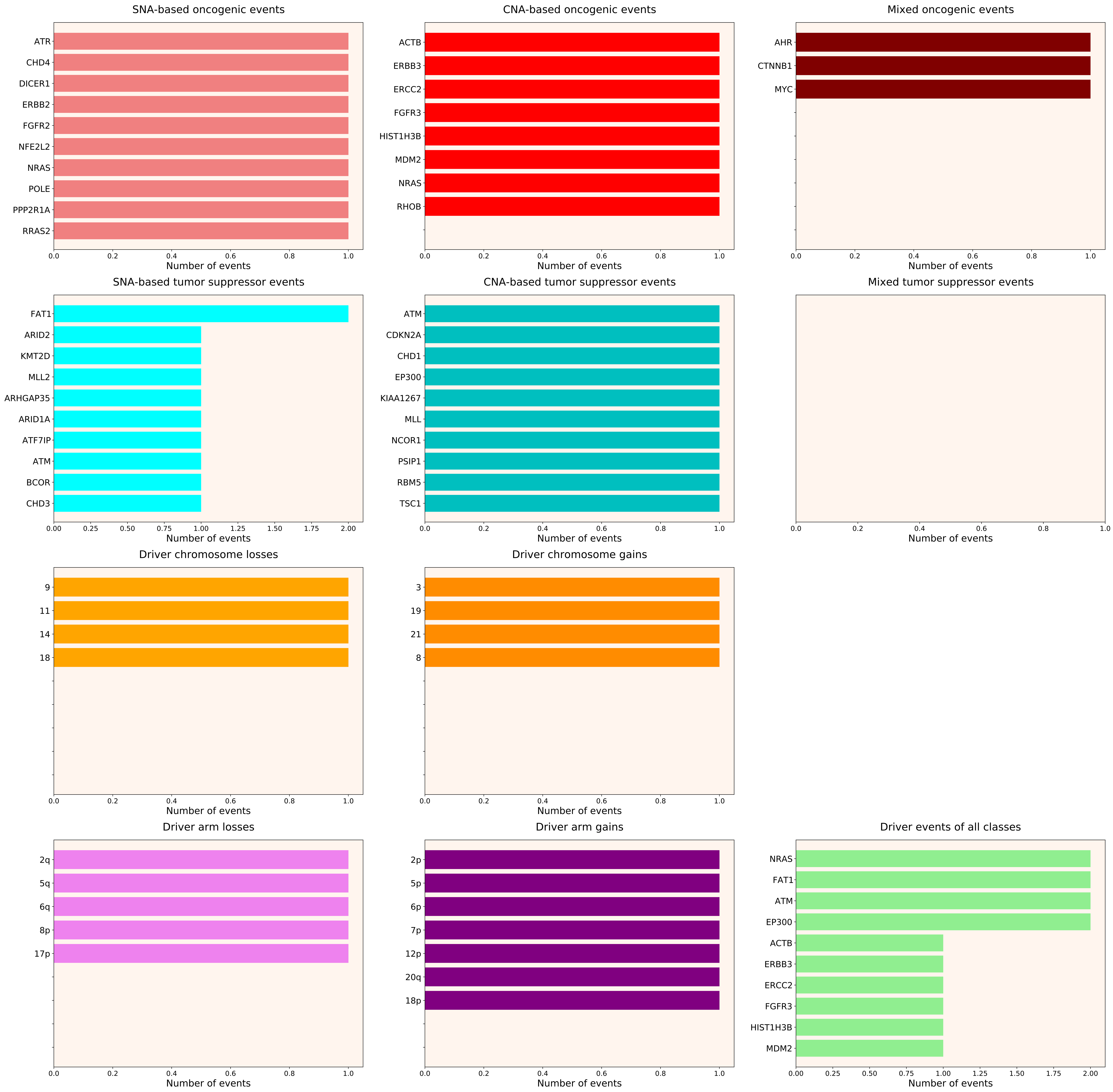
SNA-based oncogenic events
ATR
CHD4
DICER1
ERBB2
FGFR2
NFE2L2
NRAS
POLE
PPP2R1A
RRAS2
Number of events
CNA-based oncogenic events
ACTB
ERBB3
ERCC2
FGFR3
HIST1H3B
MDM2
NRAS
RHOB
Number of events
Mixed oncogenic events
AHR
CTNNB1
MYC
Number of events
SNA-based tumor suppressor events
FAT1
ARID2
KMT2D
MLL2
ARHGAP35
ARID1A
ATF7IP
ATM
BCOR
CHD3
Number of events
CNA-based tumor suppressor events
ATM
CDKN2A
CHD1
EP300
KIAA1267
MLL
NCOR1
PSIP1
RBM5
TSC1
Number of events
Mixed tumor suppressor events
Number of events
Driver chromosome losses
9
11
14
18
Number of events
Driver chromosome gains
3
19
21
8
Number of events
Driver arm losses
2q
5q
6q
8p
17p
Number of events
Driver arm gains
2p
5p
6p
7p
12p
20q
18p
Number of events
Driver events of all classes
NRAS
FAT1
ATM
EP300
ACTB
ERBB3
ERCC2
FGFR3
HIST1H3B
MDM2
Number of events
